# Nanocellulose Production: Exploring the Enzymatic Route and Residues of Pulp and Paper Industry

**DOI:** 10.3390/molecules25153411

**Published:** 2020-07-28

**Authors:** Michele Michelin, Daniel G. Gomes, Aloia Romaní, Maria de Lourdes T. M. Polizeli, José A. Teixeira

**Affiliations:** 1 CEB—Centre of Biological Engineering, Universidade do Minho, Campus Gualtar, 4710-057 Braga, Portugal; mimichelin@ceb.uminho.pt (M.M.); aloia@ceb.uminho.pt (A.R.); jateixeira@deb.uminho.pt (J.A.T.); 2 Department of Biology, Faculdade de Filosofia Ciências e Letras de Ribeirão Preto, Universidade de São Paulo, Ribeirão Preto SP 14040-901, Brazil; polizeli@ffclrp.usp.br

**Keywords:** nanocellulose, enzymatic hydrolysis, cellulases, eucalyptus Kraft pulp, biorefinery

## Abstract

Increasing environmental and sustainability concerns, caused by current population growth, has promoted a raising utilization of renewable bio-resources for the production of materials and energy. Recently, nanocellulose (NC) has been receiving great attention due to its many attractive features such as non-toxic nature, biocompatibility, and biodegradability, associated with its mechanical properties and those related to its nanoscale, emerging as a promising material in many sectors, namely packaging, regenerative medicine, and electronics, among others. Nanofibers and nanocrystals, derived from cellulose sources, have been mainly produced by mechanical and chemical treatments; however, the use of cellulases to obtain NC attracted much attention due to their environmentally friendly character. This review presents an overview of general concepts in NC production. Especial emphasis is given to enzymatic hydrolysis processes using cellulases and the utilization of pulp and paper industry residues. Integrated process for the production of NC and other high-value products through enzymatic hydrolysis is also approached. Major challenges found in this context are discussed along with its properties, potential application, and future perspectives of the use of enzymatic hydrolysis as a pretreatment in the scale-up of NC production.

## 1. Introduction

Nowadays, there is an increasing interest in the sustainable production of biofuels, chemicals, and materials from lignocellulosic biomasses due to growing environmental concerns related to the use of non-renewable fossil resources. In fact, reaching this target is one of the most challenging issues faced by our society, being a required step in the transition towards a cleaner development based on a circular economy. In this context, cellulosic ethanol production has been the most important driving force for the development of lignocellulosic biorefineries in the last decades. Nevertheless, lignocellulose-to-ethanol production is still not economically viable due to the high initial investment and elevated cost of production related to the deconstruction of the recalcitrant structure of lignocellulosic materials.

To tackle this limitation, the scientific community made huge efforts on the production of value-added products from other fractions (namely, hemicellulose and lignin) coupled with efficient ethanol production. In fact, several lignocellulosic biorefinery schemes have been proposed in the literature, in which cascading processes are suggested for the development of multiproduct facilities. Generally, these processes involve the fractionation of the lignocellulosic biomass, which usually requires chemical or physical pretreatments to recover the main macromolecules (cellulose, hemicellulose, and lignin) facilitating the access of enzymes (i.e., cellulases) to cellulose and the subsequent enzymatic hydrolysis prior sugar fermentation into biofuels or other value-added compounds (i.e., lactic acid, succinic acid, etc.). In this sense, most of the studies are focused on the disruption of the recalcitrant structure of cellulosic materials. Cellulases are able to hydrolyze the amorphous regions of cellulose about 30 times faster than crystalline regions [1]. For this reason, the solid fibrous residue resulting from cellulosic ethanol processes using an enzymatic hydrolysis route is composed mainly by crystalline cellulose [1,2].

In addition to the interest in cellulose as a putative source of fermentable sugars, cellulose is a well-known material used for a long time in the manufacturing of a wide range of products and materials [3]. Recently, the production of cellulose at a nanoscale (known as nanocellulose (NC)), used for the development of novel materials, has received much attention due to its interesting properties [4]. In fact, this issue has been already addressed in a large number of publications and patents [5,6,7]. Most of these studies have focused on NC production, its characterization, and the utilization of these nanostructures in nanocomposites, coatings, and films, or even in medical applications [8,9]. These nanostructures are classified in cellulose nanocrystals (CNCs) and nanofibers (CNFs) and can be typically obtained using strong acid and mechanical treatments, respectively. The production of NC using strong acid hydrolysis, though, is not compatible with a biorefinery scheme, since costly steps of neutralization are required previous to the fermentation process, and other thermochemical processes (such as pyrolysis, liquefaction, and gasification) are not possible due to the high degree of depolymerization of macromolecules into monomers [10]. Therefore, the enzymatic hydrolysis route to obtain NC is shown as a promising alternative to chemical catalysis, as it is compatible with biological processes and yields nanostructures easier to functionalize and with high thermal stability [11]. The use of enzymes in processes of NC production is less explored in the literature comparatively to chemical and mechanical processes. Nevertheless, several authors have evaluated this strategy in combination with other treatments and/or integrated with the production of biofuels [2,11,12].

Despite the numerous works reviewing NC production and their multiple applications [6,7], the enzymatic route is rarely approached. Ribeiro and co-workers [13] have recently reviewed the enzymatic pathway for NC production, focusing on the characterization methods and the catalytic mechanism. On the other hand, Pirich et al. [14] recently published a review dedicated to enzyme-based strategies to produce nanocellulose.

Taking all the above into account, this review aims to provide an overview of the sources of cellulose to produce NC, comparing the chemical/mechanical and enzymatic processes. In addition, the description of the main enzymes involved and their action mechanism, including the main advantages and drawbacks, are also described and discussed, as well as the main properties of the nanoscale cellulose. Moreover, the integrated production of NC and second-generation ethanol or other value-added compounds following the biorefinery concept is also approached. To conclude, the market, possible applications, and the main challenges related to enzymatic hydrolysis for NC production are also addressed.

## 2. Cellulose Structure and Types

Cellulose is the most abundant polymer worldwide with a production of 7.5 × 10^11^ ton/year and is the main component of plants, placed in the cell wall. Chemically, cellulose is constituted by a linear homopolysaccharide of β-d-glucopyranose monomers linked by β-1,4 glycosidic bonds, achieving a degree of polymerization (DP) between 10,000–15,000, which is dependent on the plant species.

**Figure 1:**
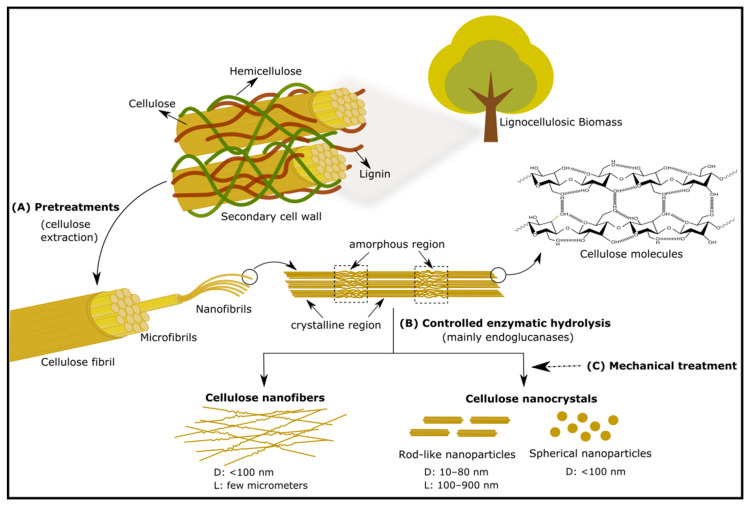
Process for nanocellulose (NC) production through enzymatic hydrolysis. (**A**) Pretreatments of the lignocellulosic biomass for cellulose extraction; (**B**) Controlled enzymatic hydrolysis for production of cellulose nanofibers and cellulose nanocrystals (rod-like and spherical) and their respective sizes; (**C**) Indication of the possible application of mechanical treatment after enzymatic hydrolysis, usually employed to obtain more uniform particles.

The three OH^−^ groups present in the anhydroglucose unit form strong intra- and intermolecular hydrogen bonding networks with the following glucose unit in the same chain and with a different chain, respectively [15]. In the crystalline region, these OH^−^ networks are strong and packed, which confers interesting features such as toughness, strength, fibrous, insoluble in water, and highly resistant to organic solvents. Therefore, its most important identified properties are: the structure forming microfibrils, the hierarchical organization (including crystalline and amorphous regions), and a glass transition temperature, higher than its temperature of degradation [16]. In nature, near to 36 molecules of cellulose are typically assembled together into elementary fibers (protofibrils), which are packed in microfibrils, being assembled into cellulose fibers [3]. Figure 1 shows the hierarchical structure of cellulose, which is composed of fibrils displaying a crystalline structure (highly ordered region) and an amorphous structure (disordered region) [15].

The native allomorph of cellulose is known as cellulose I, which can be transformed into other polymorphs or allomorphs, depending on the plant source and the chemical or thermal treatments used for their extraction. Therefore, identified polymorphs of cellulose are cellulose I, II, III_I_, III_II_, IV_I_, and IV_II_ [3]. Cellulose type I arises in two allomorphs, I_α_ and I_β_. On the other hand, the cellulose type II is obtained after re-crystallization using aqueous NaOH [16], or it can also be produced by mercerization, which aims to swell native cellulose in concentrated NaOH solutions, removing the swelling agent after that [3]. Compared to cellulose I (that runs in parallel chains direction), cellulose II (that has antiparallel chains packing) shows a more stable structure. Cellulose III_I_ and III_II_ are obtained from ammonia (gas or liquefied) treatment of cellulose I and II, respectively. Cellulose III_II_ is characterized by a disordered phase of cellulose. Otherwise, cellulose IV_I_ and IV_II_ are prepared using cellulose III_I_ or III_II_, respectively, by heating treatment up to 260 °C in glycerol. As cellulose III, cellulose IV can be reverted to structures I or II in high temperature and wet environment [3].

## 3. Processing of Lignocellulosic Biomass for Cellulose Extraction

### 3.1. Main Source of Cellulose and Industrial Process of Extraction

Different classifications exist concerning putative raw materials as sources of cellulose [17]. Among them, the most interesting classification from an environmentally-friendly point of view divides the sources of cellulose into [6]: (i) primary, in which the cellulose is the main product of the industrial activity, including fibers for textile or paper, wood for building or energy crops for biofuels production, such as cotton, flax fibers, hardwood, and softwood pulps; (ii) secondary, that includes non-processed byproducts from industrial processes of transformation (such as rice husks, corncob, wheat straw, wood bark, oilseed rape, etc.); and (iii) tertiary, which considers residues obtained from the use and/or transformation processes, such as pulp, newspapers, sugarcane bagasse, paper sludge or agri-food wastes (e.g., tea residue).

Traditionally, the pulp and paper industry is the most important supplier of cellulose for NC production, using delignified and bleached pulps [7]. Moreover, bleached pulp wastes from this industry are more easily transformed into NC, reducing the cost of the process and the consumption of chemicals and energy [18]. Therefore, researchers started to work directly using purified cellulose for NC production, such as microcrystalline cellulose or bleached Kraft pulp [4]. The main species used for cellulose extraction include hardwoods (such as eucalyptus, birch, aspen, poplar), softwoods (pine, spruce, fir, larch, hemlock), as well as agro-industrial residues (sugarcane bagasse, wheat, and rice straws) [6,19]. The industrial manufacturing process for cellulose pulps involves the preparation of raw material, the pulping process and bleaching. During the pulping step, wood is reduced to wood chips, and lignin is solubilized to obtain cellulose fiber using several chemical methods: (i) Sulfite pulping consists of a process where the wood chips are treated with H_2_SO_3_ and HSO_2_^−^ ions to disrupt lignin from cellulose fibers. (ii) For the Kraft process, the wood chips are submitted to NaOH and NaS_2_ treatments using high temperature and pressure to remove lignin from cellulose. (iii) In the soda process, which is the most employed process for agro-industrial residues, and NaOH at high temperatures is used to solubilize lignin from cellulose fibers [20]. In addition, chemical-mechanical pulping combines the chemical and mechanical refining treatments, such as wood cooking with NaOH to remove lignin from cellulose fibers, and then they are submitted to mechanical treatment. After that, the use of enzymes (namely, xylanases and laccases) has been increasingly employed as an environmentally friendly strategy to eliminate the remaining lignin in the pulp and to reduce the chemicals consumption in the process [21]. Finally, the bleaching process consists in the complete removal of lignin using chemical, gases, and steam, to provide the required color for paper manufacturing.

### 3.2. Alternative Sources and Methods for Cellulose Extraction

In addition to cellulose pulps from wood, industrial processed and non-processed sources of cellulose, including wastes generated in the pulp and paper industries (namely, bark, chips, sawdust and sludge), are shown as interesting and attractive raw materials for NC production [6,22]. In fact, the selection of renewable materials as an alternative to non-renewable resources is required to achieve sustainable processes according to European directives. The minimization of residues by using environmentally friendly processes to produce renewable materials such as NC is in line with Agenda 2030 to achieve sustainable development. Moreover, this approach can provide economic benefits, increasing the industrial value chain [23]. Therefore, the evaluation of byproducts and wastes as a source of cellulose is also considered by industries and the scientific community [24].

As mentioned above, the most important pretreatments required for purified cellulose extraction, independently of the lignocellulosic biomass source, are the delignification or pulping and the bleaching for cellulose purification. The delignification process (or pulping) aims to remove non-cellulosic fractions based on an alkaline treatment with NaOH or KOH and mineral acids, such as acid-chlorite treatment. Nevertheless, these processes are usually improved using other non-chemical treatments (such as ultrasounds or microwaves) [6], when mainly agro-industrial residues are used. For instance, Espinosa and co-workers [25] used soda pulping (100 °C, 150 min, and 7% of soda) for cellulose extraction from wheat straw, which increased α-cellulose up to 62%, decreasing the content of hemicellulose, lignin, and extractives. For improving the nanofibrillation of a semi-chemical soda pulp, three pretreatments (namely, mechanical treatment using a screw extruder and a high-pressure homogenizer, and enzymatic hydrolysis treatment with endoglucanases) were evaluated showing a significant decrease on the degree of polymerization of cellulose.

As an alternative to the traditional delignification process mentioned above, the use of advanced cascading conversion technologies, which enables the application of a biorefinery approach, is also desirable [18]. For instance, apple tree pruning and pea stalks were used for cellulose CNCs production after being pretreated by autohydrolysis (for hemicellulose solubilization) and organosolv-acetosolv (60%, 180 °C, for 90 min), as an alternative to conventional NaOH pretreatment. This study showed a significant effect of pretreatment on CNCs properties, in which organosolv and autohydrolysis were more appropriated to obtain more homogeneous and smaller size particles than NaOH treatment [18]. Liquid hot water (120 °C and 1 atm) was also employed for the extraction of cellulose fibers from plum seed shells and compared to alkali treatment with NaOH [26]. On the other hand, hydrothermal pretreatment combined with an organosolv process was also evaluated for sugarcane bagasse delignification, and it was refined by Bauer disk, yielding a pulp with 92% of cellulose that was used to produce nanofibrillated cellulose films employing an enzymatic treatment followed by high-intensity sonication [27]. Other alternative solvents, such as [Bminm]Cl ionic liquid, assisted by microwave heating (130 °C, 500 W) were used for cellulose extraction from sugarcane bagasse [28]. In addition, attention is also on the use of more environmentally-friendly solvents, such as the study described by Kwok and collaborators [29], who developed a methodology for the screening of less hazardous solvents for extracting high-value lignin before Kraft pulping to obtain cellulose. As described for the pulp and paper industry, the bleaching aims to completely remove lignin using chlorine in an acid media and to reduce the diameter of cellulose fibers, improving their features (including surface area, crystallinity, and aspect ratio). As for the pulping step, alternative processes for pulp bleaching have also been proposed (using hydrogen peroxide or ozone) to avoid the use of chlorine in this process. Recent technological developments in ozone-based pulp bleaching have been reviewed by Tripathi and collaborators [30]. Taking into account the different alternatives for cellulose extraction, the properties of cellulose nanostructures do not depend only on factors such as plant species, growing conditions, and part of the plant, but also the pretreatment used for the lignocellulosic biomass processing has demonstrated to have a significant influence on the mechanical and thermal performances, the crystallinity, morphology, and surface charge [6].

Regarding cellulose sources derived from the pulp and paper industry, most of the works collected in the literature refer to the use of Kraft pulps. Nevertheless, other processed residues from this industry, such as waste paper, which is composed mainly of cellulose (60–70%), has also been used for NC production [22]. This residue is considered a promising source of cellulose since a previous treatment for cellulose extraction is not required. In fact, wastepaper is shown as a raw material with high potential since harsh conditions to produce NC are not necessary, which can yield up to 64% depending on the wastepaper used. Moreover, recycled paper mill sludge was also already evaluated for NC production [31]. Other byproducts from the pulp and paper industry, such as wood bark that still contains 45% of cellulose [32,33], could also be an interesting raw material to be evaluated.

## 4. Nanocellulose Classification

**Figure 2:**
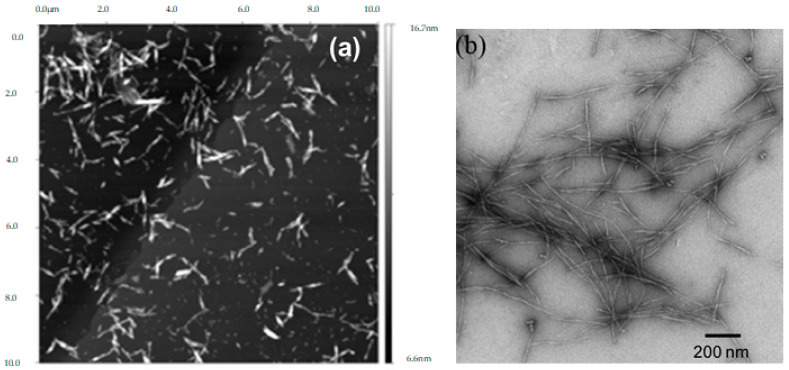
Images of cellulose nanocrystals (CNCs) by AFM (atomic force microscopy) (**a**) and cellulose nanofibrils (CNFs) by TEM (transmission electron microscopy) (**b**) obtained by enzymatic hydrolysis followed by mechanical treatment. Reprinted from [35] and [36], respectively, Copyright (2020), with permission from Elsevier.

Nanostructures of cellulose are comprised by cellulose nanocrystals (CNCs) and cellulose nanofibrils (CNFs) (Figure 2), which are generally obtained by acid hydrolysis treatment and mechanical processing, respectively. In addition to these designations, other synonyms can be founded in literature. For CNCs, it is also used for nanocrystalline cellulose (NCC), crystallites, (nano)whiskers, and rod-like cellulose microcrystals. On the other hand, for CNFs, it can also be designated for microfibrillated cellulose (MFC), nanofibrillated cellulose (NFC), and cellulose microfibrils (CF) [4,34].

Typically, the method for CNCs production involves controlled acidic digestion with H_2_SO_4_ of the amorphous regions of cellulose, first reported in 1951 by Ränby [3]. During acid hydrolysis, the amorphous regions are preferentially hydrolyzed, while crystalline regions present a higher resistance to acids attack [9]. After acid treatment, CNCs are washed with water to stop the reaction and are recovered by centrifugation. Then, dialysis is employed to remove free acid molecules, and a mechanical process (namely, sonication) is used to disperse the nanocrystals and obtain a stable suspension. Finally, concentration and drying of the suspension can also be carried out [9]. CNCs can be obtained from several cellulose sources and employing distinct methods; however, the use of wood or cotton (as the main source of cellulose) and acid hydrolysis with H_2_SO_4_ are still the most common routes for CNCs production [24]. Nevertheless, the use of strong concentrated acids is corrosive and incompatible with an environmentally-friendly process [9].

Regarding the production of CNFs, the delignified and bleached pulps are submitted to mechanical treatments, which includes equipment such as high-pressure homogenizers or microfluidizers, ball millings, steam explosion reactors, high-speed blenders, extruders, and ultrasonic equipment [7]. The main disadvantage here is the high energy consumption. Therefore, several works showed a reduction of energy requirements when mechanical processes are combined with enzymatic and/or chemical treatments (such as TEMPO (2,2,6,6-tetramethylpiperidine-1-oxyl) oxidation and carboxymethylation) [7,37,38,39,40].

Table 1 presents the NC production yield as well as the amount of chemicals and enzymes estimated from different production reports on literature, for a 100 kg base of raw material.

## 5. Nanocellulose Production by Enzymatic Hydrolysis

The utilization of enzymes in the production of NC is rather complex and may refer to very distinct roles in the overall process, ranging from removing pectins and hemicellulose to the most common application of cellulases to produce NC. The focus of this review, however, will be the action of enzymes on the production step.

Despite the still common idea that CNCs are generally produced from acid hydrolysis while CNFs are produced from mechanical fibrillation (assisted by other processes), numerous studies have been conducted showing that the mechanisms for producing both materials are in fact more diverse; multiple works refer to the application of cellulases for the production of CNCs combined with acid hydrolysis or even with no chemicals being used (Table 2).

### 5.1. Different Approaches for the Integration of Enzymatic Hydrolysis on Nanocellulose Production

Table 2 lists some previous studies addressing the extraction of NC through an enzymatic process. Distinct process configurations have been used depending on different factors, such as the source of cellulose or the type of material being produced. In general terms, this process involves two main phases: the extraction of cellulose (see Section 3) and the production of NC. Studies reporting the utilization of raw materials, which still did not undergo any kind of processing, normally involve an initial mechanical (e.g., grinding, milling) and/or physicochemical (e.g., autohydrolysis, alkali, bleaching) pretreatment to extract cellulose (eliminating hemicellulose, lignin, and other components). Opposed to that, highly processed materials, such as bleached Kraft pulps [43,44,45], cotton linters [42,46] or microcrystalline cellulose [47], have no need for this step; typically, in these cases, only a minor preparation step is conducted, such as dispersing or swelling of the cellulosic material [48]. This is followed by the process of enzymatic modification of the cellulose-enriched solid, which will introduce different types of modification according to the enzymes being used.

In the particular case of CNFs production, the enzymatic hydrolysis is commonly followed by a mechanical step of fibrillation, such as homogenization [38,44], microfluidization [49,50], extrusion [25,51], and sonication [27,52], among others. Few studies have also reported no mechanical treatment step after hydrolysis [12,53,54,55], hence totally relying on the enzymes to produce the nanofibers.

Regarding the extraction of CNCs, the most common procedure consists on a sonication step after hydrolysis, allowing an intense fragmentation of NC into particles within a nanoscale size range [62] and their efficient dispersion, even though some authors have also described its use before hydrolysis [67]. Some studies have equally reported the application of sulfuric acid [42,68] or other chemicals [2] after enzymatic hydrolysis or, in total opposite, no treatment after enzymatic hydrolysis [11,48,69,70,71].

Either for CNFs or CNCs, it should be noted that there is no established procedure for their production, being instead of a case-specific approach that will largely depend on the initial raw material and the required properties for the final material.

### 5.2. Mechanisms of Cellulolytic Enzymes

**Figure 3:**
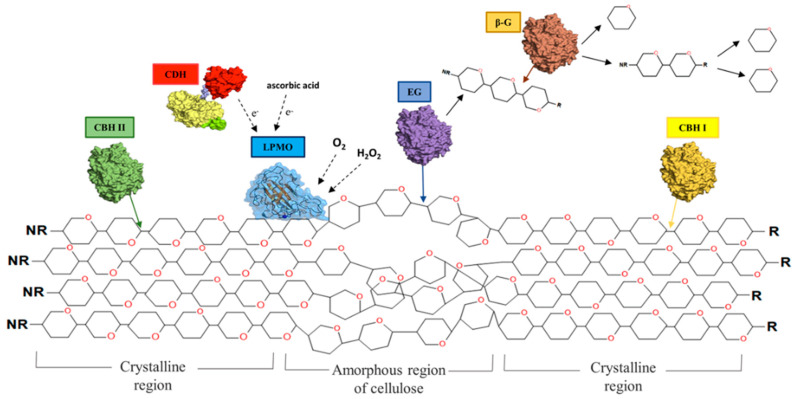
Overall schematic representation of the mechanisms involved in NC production by distinct classes of enzymes (adapted from [72]). R: reducing cellulose end; NR: nonreducing cellulose end; EG: endoglucanase; CBH I: exoglucanase removing cellobiose from R end; CBH II: exoglucanase removing cellobiose from NR end; β-G: β-glucosidase; CDH: cellobiose dehydrogenase; LPMO: lytic polysaccharide monooxygenases.

Even though different classes of enzymes have been used for the extraction of NC, cellulases correspond to a large majority of these cases. Figure 3 presents an overall scheme of the enzymes involved in NC production. Their ability to attack cellulose fibers with a high selectivity enables different types of modifications over cellulose. Cellulases are a specific class of enzymes that directly catalyze the hydrolysis of cellulose into simpler sugars, hence being commonly produced by cellulolytic organisms such as those from the genera *Clostridium*, *Trichoderma*, and *Aspergillus*, among others [14].

#### 5.2.1. The Cellulolytic Complex—Synergetic Deconstruction of Cellulose

Cellulolytic organisms typically code for and secrete a large number of distinct cellulases, which, according to their specific mechanism, can be assigned into two main categories. Endoglucanases (EGs) cleave random internal β-1,4-glycosidic bonds of the cellulose chains, typically on the amorphous regions, originating new cellulose chain ends; cellobiohydrolases (CBHs), or exoglucanases, processively act on the different extremities of cellulose chains forming cellobiose units. One final class of cellulases refers to β-glucosidases, which hydrolyze cellobiose into glucose [73].

#### 5.2.2. The Major Role of EGs: Single Component vs. Enzymes Mixture

Comparatively to the application of cellulases for the hydrolysis of lignocellulosic biomass, the production of NC represents a distinct paradigm. Either for CNCs or CNFs, cellulases would only require acting to a given extent and over specific sites of cellulose, providing the desired level of modification over cellulose structure. Opposed to this, cellulases cocktails for biomass hydrolysis aim for maximum conversion of complex sugars into fermentable ones [43]. Specifically, for producing CNCs, the internal cellulose amorphous regions are the main target; these are hydrolyzed by EGs, resulting in smaller chains of crystalline cellulose. Something similar occurs for CNFs, although total removal of amorphous regions is not intended here, but instead, a reduction of cellulose chain length and the cleavage of inter-fiber linkages, which critically enhance posterior mechanical fibrillation. While in both cases EGs provide a desirable action towards a reduction in fiber length (DP) or an increase of crystallinity, the action of CBHs may result in undesirable fiber digestion into cellobiose. This fact highlights the important role of the composition of the enzymatic mixture on the final yield of extraction, raising the question of whether a complex cellulases cocktail may be the most suitable option in this context. In fact, even though several studies report the use of complex cocktails, such as Cellic CTec [2,41,55], the most frequent option refers to the application of monocomponent endoglucanase, such as the Novozym 476 [40,49,50], preparations for fibers modification, such as Fibercare R [36,62,74] and Cellusoft L [75,76], or other individual EGs [45].

While an extended action of CBHs will result in a decrease of cellulose fibers [14], their action can be partially constrained by an accumulation of cellobiose through a mechanism of end-product inhibition [14], which would be favorable to prevent thorough enzymatic degradation. This would especially happen when low levels of β-glucosidase are present, which possibly explains numerous works employing Celluclast for NC production [27,36,47,65].

### 5.3. Other Enzymes in Nanocellulose Production

Adding to the typically used cellulases, the process of NC production can be complemented with the application of other enzymes, either to provide a more purified cellulose material or to assist cellulases action.

#### 5.3.1. Xylanases

After cellulases, xylanases is the most common class of enzymes applied for NC production. Acting on xylan residues, these enzymes can reduce the barrier formed by this polymer to cellulases action, significantly improving the extraction process [52]. When Song et al. [77] supplemented Cellic CTec2 with HTec2 in the hydrolysis of northern bleached hardwood Kraft (NBSK), glucose production increased approximately 40%, even though only trace amounts of xylose were produced. According to Hu et al. [78], xylanases may also act synergistically with cellulases improving fibers’ swelling and porosity, therefore increasing cellulases’ accessibility to cellulose. On the other hand, partial/total elimination of xylan residues can also affect the final properties of NC. According to Siqueira et al. [45], the presence of xylan in the final nanomaterial can increase its colloidal stability since it affects fibers’ aggregation. This may be attributed to the fact that xylan can act as an obstacle hindering the irreversible binding of hydroxyl groups on different cellulose fibers [79]. On a previous study of Winter et al. [80], the authors inclusively observed that the addition of xyloglucan to the produced CNCs improved its colloidal stability.

Xylanases can be used following different approaches, according to the initial raw material and the final expected product. Since enzymes are normally used to complement other chemical or mechanical treatments, the exclusive utilization of xylanases would be ultimately sufficient to produce some NC materials, as the removal of xylan already facilitates mechanical fibrillation. Tibolla et al. [12,57,81] reported the utilization of xylanase (from Novozymes) to produce CNFs from banana peel solid, while Hassan et al. [82] described the production of MFC from date palm fruit stalks using commercial xylanase from *Thermomyces lanuginosus*. This, however, cannot be totally accomplished as cellulases and xylanases are usually found in each other’s cocktails, even in trace amounts; ranging levels of xylanase activity have been reported in Cellic CTec2 [83], Celluclast [78], and Viscozyme [84], while Cellic HTec2 has also been reported to present some FPase and CMCase activities [83]. Consequently, on a low-hemicellulose solid scenario, the trace amounts of xylanase activity in the cellulase cocktail would possibly be enough for substantial hemicellulose hydrolysis [41,55,85]. Opposed to that, combined action of a cellulase and hemicellulase mixture may be advised when hemicellulose is present in higher amounts. Establishing the adequate levels of xylanase activity will be case-specific, as it would be based on the solid composition (including the presence of lignin) but also the desired properties for the final material. As an example, Chen et al. [86] observed that using a complex mixture of cellulases and xylanases allowed performing faster and with higher efficiency compared to exclusively cellulases, however, excessive use of xylanase did not lead to an increase of the extraction yield. On the other hand, in a recent study by Tong et al., [48], the authors observed that the ratio of cellulases-xylanases had a clear effect on final NC morphology. For a cellulase concentration superior to xylanase the final CNCs presented a spherical form with an average diameter ranging from 40–70 nm. When xylanase concentration increased above the concentration of cellulase the CNCs clearly changed into a rod-like morphology, with their length increasing with the increase of xylanase (750–1000 nm).

#### 5.3.2. LPMOs

Another class of enzymes that may assist NC production are the lytic polysaccharide monooxygenases (LPMOs), which have been increasingly used on cellulase cocktails employed for lignocellulosic hydrolysis. LPMOs act on cellulose chains by oxidative cleavage of glycosidic linkages, leading to the formation of oxidized glucose units in different positions, and consequently, a substrate more susceptible to hydrolysis by cellulases [87]. As for cellulases, LPMOs are typically secreted by saprotrophic fungi when grown on cellulose or a lignocellulosic material [88,89]. In the specific context of NC production, few studies have reported on the utilization of LPMOs. One example refers to the work conducted by Hu et al. [52] who studied the potential of combining individual endoglucanases with other enzymes, such LPMOs, to improve the fibrillation of a bleached Kraft pulp and/or the properties of the final material. Even though major differences were not found over the dimensions and morphology of the final fibers, some intrinsic properties were improved. LPMO family AA9 was found to increase the negative charge of cellulose fibers, possibly due to deposition of carboxylic acid or ketone structures resulting from the oxidative cleavage. According to the authors, this increase may have contributed to a significant rise of ζ (zeta) potential, aiding cellulose nanofibrillation and leading to a more stabilized suspension. In addition, in a recent study from Valls et al. [90], the authors observed that the addition of a bacterial LPMO to the cellulases mixture had numerous effects over NC production from cotton linters. LPMOs addition led to a significant increase in the amount of sugars released during extraction (~2-fold), the highest yield of extraction after mechanical fibrillation (23%) and the lowest value of fibers length.

### 5.4. Relevant Factors on the Enzymatic Process

Similar to any enzyme-mediated process, NC production can be affected by several parameters; some of them directly influence cellulases efficiency (e.g., pH, temperature, binding affinity), while others can dictate the way enzymes will act over cellulose, hence affecting the yield of the process and the properties of the final NC.

#### 5.4.1. Raw-Material Composition and Pretreatment

The influence of the raw material on NC production occurs over different levels. A first issue to consider is the purity of the solid before enzymatic hydrolysis, which will rely on its initial composition and the application of efficient methods to remove non-cellulosic components (e.g., hemicellulose, lignin, extractives, protein). Since enzymatic hydrolysis almost exclusively changes cellulose (some enzyme preparations also have trace levels of xylanase activity), using a less purified solid will lead to final NC particles with more impurities. In this context, the presence of substantial amounts of lignin can represent a special challenge since cellulases have a reportedly high affinity to this component through irreversible binding [91]; the non-productive binding of cellulases is a major issue of lignocellulosic hydrolysis, leading to higher consumption of enzymes and a consequent increase in the process cost [92]. The real extent of it will depend on the amounts of lignin, but also its chemical structure, as lignin–cellulase interactions are mainly hydrophobic [93]. Adding to this, lignin may equally form a physical barrier to holocellulose in the substrate, critically affecting enzymes accessibility [94].

A most relevant aspect to discuss is also the distinct natural affinity of enzymes for different solids [95] or, on the other hand, the fact that distinct enzyme preparations may present diverse solid–enzyme interaction behaviors for the same solid [96]. It is widely known that enzyme-substrate interaction is a very complex mechanism that can be significantly affected not only by environmental factors (e.g., pH and surfactants), but also numerous structural properties of both enzymes and solids [97]. To a great extent, this may result from the specific affinity of carbohydrate-binding modules (CBMs), normally found in fungal cellulases, to different types of solids. In a previous work by McLean et al. [98], the authors observed that a CBM presented different affinities to the solid according to its crystallinity. Another point to consider is the possible influence of structural properties of the solid in the way enzymes will act over cellulose. As pointed out by Brinchi et al. [9], solids with high crystallinity, such as tunicate or bacterial cellulose, have an inferior fraction of amorphous regions that will be cleaved, which may result in the formation of larger CNCs.

#### 5.4.2. Enzyme Dosage and Hydrolysis Time

Adding to the composition of the enzyme’s mixture, the time of hydrolysis and the enzyme dosage are possibly the most important factors for the controlled and selective production of NC. On the basis of the well-established mechanisms of EGs and CBHs, it would be expected that employing different concentrations of the enzyme would, for the same period of time, result in different NC products.

In an early report from Chen et al. [67], the authors observed that while the levels of reducing sugars increased for growing enzyme dosages (10–19 FPU(filter paper units)/g), the yield of CNCs reached a maximum at 13 FPU/g, decreasing for higher levels of the enzyme. The same behavior was observed in what concerns the time of hydrolysis, where a period of two days was found as the optimum time of hydrolysis. In a more recent study from Tong et al. [48], both the enzyme concentration and the hydrolysis time showed to clearly influence the morphology of CNCs. The utilization of lower levels of an enzyme (1–20 U/mL) resulted in rod-like CNCs with a length/diameter in the range of 600–800 nm and 20–40 nm, respectively. When enzyme concentration further increased to 50 U/mL, a new spherical form of CNCs appeared, with an average diameter of 50 nm, although coexisting with the rod-like morphology; a significant increase in enzyme concentration over levels of 300 U/mL, together with a reduction on the hydrolysis time to 5 h, led to CNCs being produced mostly in the spherical form. Similarly, under a low enzyme dosage (10 U/mL) the CNCs presented a rod-like morphology with a length decreasing from 800–900 nm for a 6 h hydrolysis, to 500–600 nm for 18 h hydrolysis. Similar observations were made by Chen et al. [64], who analyzed the CNCs produced under a range of operational times and enzyme dosages. For 5 h hydrolysis, the utilization of an enzyme dosage in the range of 10–50 μ/mL resulted exclusively in ribbon-like CNCs, with their dimensions decreasing with the amount of enzyme; an increase of the enzyme levels to 100–300 μ/mL led to the emergence of a granular form, becoming the exclusive morphology for the highest enzyme concentration. According to the authors, this seems to suggest that, contrary to the well-established idea that EGs only attacks internal amorphous regions of cellulose chains, under high-concentrations of enzymes they may also attack the crystalline regions, a process that can be especially favored with a previous swelling of the fiber. Furthermore, using an enzyme dosage as low as 20 μ/mL, the length of the ribbon-like CNCs gradually decreased from 400–500 nm using hydrolysis of 5 h, to 250–400 nm for hydrolysis of 11 h, which highlights the possibility for rigorous control of CNCs morphology with these parameters.

#### 5.4.3. Temperature and pH

Like all enzymes, cellulases optimally operate in a given range of pH and temperature values. Furthermore, these are associated with intrinsic properties of each enzyme, which may result in irreversible enzyme loss when operating in extreme conditions. Typically, cellulolytic enzymes present an optimum performance for a temperature ranging from 40–50 °C and a pH between 4 and 5 [99], which correspond to relatively mild operation conditions comparatively to the alternative chemical treatments.

A controlled pH during enzymatic extraction is particularly important since it will affect the adsorption/desorption of enzymes over cellulose [97], and consequently, their hydrolysis efficiency. A previous study from Rodrigues et al. [100] showed that when the pH increased from 4.8 to 9–10, significant conformational changes occurred in the structure of Cel7A; these, however, were reverted when pH was restored to 4.8. This fact may suggest that the exposure of cellulases to unusual values of pH has clear effects, but these can be reverted in some cases, which would mean no loss of enzyme. In the specific context of NC production, the common pH range is 4–5.8, with only a few exceptions; both Li et al. [46] and Paakko et al. [63] already reported the utilization of a pH of 7. This range of pH values is especially relevant for the cases when enzymatic treatment is preceded by an alkaline [11,101] or acidic [65,102] treatment, intended for hemicellulose/lignin removal, or the first step of NC production; this would require intensive washing and/or pH adjustment (e.g., dialysis) prior to the addition of enzymes.

As for pH, the temperature range on enzymatic NC production is also very narrow, usually between 45 and 55 °C; some exceptions refer to non-fungal enzymes or the direct use of a microbial consortium. In a previous work by Teixeira et al. [103], CNCs were enzymatically produced using a blend of an EG and a β-glucosidase from *Pyrococcus horikoshii* and *Pyrococcus furiosus*, respectively, both hyperthermophilic; consequently, the hydrolysis temperature was 85 °C. In addition, Ma et al. [70] reported the utilization of the commercial preparation Giant A at 60 °C, in accordance to its optimal temperature. Referring to the utilization of a microbial consortium (enriched in cellulase producers), Satyamurthy and Vigneshwaran [104] employed a hydrolysis temperature as low as 35 °C. In a total distinct situation from what occurs for pH, temperature control will be even more critical. While operating under the optimum temperature range will lead to reduced hydrolysis, a substantial increase over this range can result in irreversible denaturation [105]. Considering how the temperature can significantly affect cellulases’ efficiency, it is surprising the lack of studies clarifying its potential effect over processes of NC production.

#### 5.4.4. Composition of the Cellulases Mixture

As for the selection of the enzyme dosage and operational time, the composition of the enzyme mixture will be equally critical for a controlled NC production. Different cellulases preparations can present different classes of enzymes (CBHs, EGs, hemicellulases, LPMOs), in different ratios and from different sources (e.g., fungal, bacterial). Accordingly, and also on the basis of their distinct selectivities, the overall synergism which one may achieve is rather complex and will result in very distinct products of hydrolysis.

A recent study by Siqueira et al. [45] showed that monocomponent EGs from different origins could present very distinct action mechanisms in the hydrolysis of bleached eucalyptus Kraft pulp (BEKP). The fungal EG from GH (glycoside hydrolase) family 7 was able to solubilize 9% of cellulose while the other fungal EG from GH family 45 hydrolyzed 5%; the bacterial EG from GH family 5 only hydrolyzed 2%. Another relevant fact was that, especially for the case of fungal enzymes, a considerable fraction of the solubilized sugars was glucose (75% in the case of GH family 7), suggesting the capacity for this EG to either act on chain ends or to hydrolyze cellobiose. While this may represent a broader enzymatic activity range, it suggests a lower specificity for the amorphous regions of cellulose, a highly desirable trait in CNCs production; this capacity, however, was almost inexistent in the bacterial EG. In the scope of the possible effects that hemicellulose can have on cellulose hydrolysis, the conversion of xylan was also inspected. The fungal EG from GH family 7 was able to hydrolyze almost 25% of the xylan (mostly into xylo-oligomers), while for the EG from GH family 45 it was below 5% and almost inexistent for the bacterial EG.

Another important work on the diversity of cellulase systems refers to the study conducted by Yarbrough et al. [10] comparing the efficiency of cellulose hydrolysis and NC production of *Trichoderma reesei* and *Caldicellulosiruptor bescii* enzyme systems. The former solubilized nearly 40% of cellulose, reaching a maximum NC yield around 30% after 24 h; *C. bescii* achieved cellulose hydrolysis above 80% with a peak NC yield of 42%. Even more interesting than the levels of NC yield was the morphology of the extraction products. Through dynamic light scattering (DLS) they observed that *T. reesei* consistently produced an NC mixture composed mostly by CNFs, but also some CNCs. On the other hand, *C. bescii* showed a gradual transition over time, from an initial bimodal distribution (consisting of CNFs and CNCs) to a single and more uniform population of particle sizes, which was an intermediate of the initial ones and corresponded to CNCs. As pointed out by the authors, this may result from very distinct mechanisms of hydrolysis found in the most prevalent enzymes of these organisms. The largely abundant CBH Cel7A of *T. reesei* processively acts on crystalline cellulose, originating shorter cellulose fibrils. Opposed to that, the largely abundant multifunctional CelA complex of *C. bescii* (containing a catalytic domain of an EG family 9 and a CBH family 48) is able to bind cellulose in multiple locations and perform localized hydrolysis [106], previously referred to as “pit digging” action, which may allow fragmenting the CNFs into CNCs.

A recent review by Pirich et al. [14] refers to another potential key element in the properties of the selected enzymes. According to the authors, the absence of CBMs, which is commonly associated to a higher affinity of enzymes to crystalline cellulose, can possibly result in inferior hydrolysis of the crystalline regions, a desirable trait in nanocellulose production.

#### 5.4.5. Assistance by Mechanical Treatment

Even though enzymatic processes were only more recently raised as a new option to complement the well-established extraction by mechanical fibrillation (CNFs) or acid hydrolysis (CNCs), numerous studies already showed that it could be used alone to produce NC materials (see Section 5.1). Despite this, adding to the initial pretreatments to obtain cellulose from raw materials (e.g., delignification), several other treatments can be employed to improve NC production or to control/optimize the properties of the final material (e.g., morphology, DP, crystallinity).

An early work from Zhu et al. [1] mentioned that the CNF sheets enzymatically produced from eucalyptus Kraft pulp had a reduction in their opacity from 93% to 24%, only after 10 passes on a microfluidizer processor (200 μm chamber). With a similar material, Qing et al. [36] observed that while the hydrolysis and refining led to a decrease in fibers’ DP from 1000 to 287, a posterior microfluidization step (15 passes; 87 μm chamber) still allowed a further decrease in the DP to 263; interestingly, the crystallinity increased during hydrolysis and refining from 55% to 60%, but decreased afterwards to 57% after microfluidization, suggesting a superior homogenization effect over the crystalline regions. Another work from Lourenço et al. [44] reported that increasing the number of passes through a high-pressure homogenizer from 2 to 6 resulted in a decrease of DP from 1834 to 1504, while the extraction yield slightly increased from 22 to 26.

Several studies also exist regarding the effects of sonication. One example consists of the work conducted by Campos et al. [62] on the production of CNFs from curauá and sugarcane bagasse. The authors observed that the diameter of fibers produced from curauá significantly decreased, from 78 μm in the raw material, to approximately 4 μm after enzymatic hydrolysis; although the bleaching before hydrolysis caused most of this reduction (to a diameter of 4.6 μm). Nonetheless, fibers with nanoscale dimensions were only obtained after the application of a sonication treatment (20 min), which reduced the diameter to 55–100 nm (depending on the enzyme mixture). This similarly occurred for fibers from sugarcane bagasse; their diameter after enzymatic hydrolysis ranged from 20–30 μm but significantly decreased to nearly 30 nm after sonication. Cui et al. [47] investigated the possible effects of applying multiple sonication steps (every 12 h) with different time durations during the enzymatic production of CNCs from microcrystalline cellulose. For a 120 h hydrolysis, the yield of CNCs production was 17.6% without sonication, increasing to 19.3% and 22.6% for a sonication step of 30 and 60 min, respectively. On the other hand, the length of the CNCs notoriously decreased from 200–400 nm, without sonication, to 100–300 nm and 50–80 nm for sonication steps of 30 and 60 min, respectively. Visible effects were also observed on the crystallinity index (CI) of the final particles: the application of a 30 min treatment led to a CI of 87.5%, opposed to 83.4% with no treatment. Interestingly, the extension of sonication to periods of 60 min led to a decrease of the CI to 82.3%, suggesting that after a specific point of fibers modification, both amorphous and crystalline regions are removed by sonication.

### 5.5. Integration of Nanocellulose Production with Other Biotechnological Products

While NC production can already be considered a high-value industry, providing diverse materials for electronics, medicine, energy storage, etc., it has been growingly associated with other compounds through an integrated production, as a mean to increase the viability of the process. A considerable fraction of NC worldwide production is, in fact, currently attributed to the pulp and paper industry [22], which saw NC as a particularly interesting co-product to improve its economic efficiency. Nevertheless, this integration can also be very relevant to the emergent concept of biorefineries, pursuing an integral valorization of the whole cellulosic material. In this sense, NC would be produced as the main product, resulting in a secondary stream of fermentable sugars which could be promptly converted into different compounds. In a different approach, the spent solid from the enzymatic hydrolysis of lignocellulosic materials, usually enriched in crystalline domains, could be used to produce CNCs. Despite this potential, literature reports on efficient integration of NC production with other value-added compounds are still scarce. This can be explained by direct competition between these classes of compounds: high production of one generally means low production of the other. In a recent study by Aguiar et al. [11] on the production of CNCs from hydrolysis of pretreated sugarcane and straw, the authors reported the production of a glucose stream with 84 and 75 g/L, respectively; the yield of CNCs production was 11.3% and 12%, respectively, markedly inferior to the levels usually obtained by acid hydrolysis, as pointed out by the authors. Bondancia et al. [55] reported the production of a glucose stream with 91 g/L and the presence of CNF structures in the final spent solid with a CI of 83%, opposed to 72% before hydrolysis. Furthermore, Camargo et al. [2] described the production of CNCs from the spent solid of the hydrolysis of sugarcane bagasse to produce fermentable sugars (complemented with acid hydrolysis). Cypriano et al. [59] reported the potential of using the citrus pulp of floaters (residue from orange juice production) to produce hesperidin, NC, and ethanol; however, NC yield was 1.4% while the maximum ethanol titers were around 2 g/L. In addition, Tsukamoto et al. [107] referred to the utilization of orange waste to produce D-limonene, NC (3% yields), and ethanol (max. 8.5 g/L). In the true sense of a biorefinery, the recent work of Guirimand et al. [108] should be highlighted since it refers to the production of NC and xylitol, which do not directly compete on their precursors. Using wood Kraft pulp (78% cellulose, 17% hemicellulose), the authors employed a consolidated bioprocessing (CBP) system, consisting of a *Saccharomyces cerevisiae* strain with cell surface display of xylan-degrading enzymes (endoxylanase and β-glucosidase), to convert hemicellulose into xylitol while enriching the cellulose content of the solid to 87%. This strategy resulted in the production of 3.7 g/L of xylitol and simultaneously enabled the nanofibrillation of the final solid residue.

While the integration of different biotechnological products may always seem interesting, it is clear that when originating from the same solid fraction, the initially expected benefits could not be that high. The optimum scenario would achieve a maximum cellulose conversion either as NC or as glucose; obtaining cellobiose or other higher molecular weight cello-oligomers is not desirable and should be avoided. In this perspective, a careful selection of the enzyme mixture employed for NC extraction/solid conversion gains especial relevance. The specific ratio of different cellulase components, with characteristically different specificities and product ranges, will dictate the maximum cellulose conversion into products of interest. In a recent study from Siqueira et al. [45] the authors analyzed three different monocomponent endoglucanases (GH family 5, 7, and 45) for NC extraction from BEKP. The authors observed notorious differences, not only in their capacity to hydrolyze cellulose (the fungal GH 7 and 45 were clearly superior to the bacterial GH 5), but also in the distribution of the final products from hydrolysis; fungal GH family 7 obtained 75% of final sugars in the form of glucose, while for the bacterial GH family 5 these were almost totally in the form of cellobiose, an unfavorable scenario to produce other products by fermentation.

### 5.6. Main Advantages of Enzymatic Nanocellulose Production

Numerous advantages can be found from the application of enzymatic processes for NC production, either comparing to other chemical treatments (e.g., sulfuric acid, TEMPO) or even to exclusively mechanical extraction.

#### 5.6.1. Operation under Milder and Less Hazardous Conditions

The first consequence from using enzymes is a partial or even total elimination of chemicals during extraction [12]; the extension of it will depend if the process is exclusively enzymatic or even aided by chemicals’ action. Accordingly, the environmental impact of the overall process will be critically reduced since the amount of effluents with a chemical load will be inferior [109]. Another immediate result from this transition is operating under milder conditions, which includes moderate temperatures, pressures, and pH [15,55]. From an operational standpoint, this can have numerous benefits, namely a safer operation, reduced costs of electricity and heat transfer utilities, and the utilization of cheaper construction materials (no requirement for anti-corrosion materials) [110,111]. In the particular context of a possible integration with a pre-established production process (e.g., cellulosic ethanol, pulp, and paper) this could also mean a smaller cost to adapt the industrial facility as less additional equipment and piping materials would be required.

An adequate approach to access these improvements would be to perform a life cycle assessment (LCA) analysis for both routes (enzymatic and non-enzymatic); but the application of this tool has still been poorly made in this context with only a few studies reported. Piccinno et al. [112] conducted an LCA analysis for the production of MFC from carrot waste by applying an enzymatic process and compared it with the results previously described by Figueirêdo et al. [113] for the production of MFC from cotton and unripe coconuts using sulfuric acid. According to the authors, the global warming potential of the sulfuric acid processes for coconut and cotton was 17.8 and 2.0 times superior, respectively, compared to the enzymatic extraction; this was mostly attributed to higher electricity consumption, which was also superior in a similar order of magnitude. Compared to the results from Li et al. [114], the global warming potential was very similar for a strategy of TEMPO-oxidation combined with homogenization but clearly superior to the other strategies (either using chloroacetic acid etherification and/or sonication). A detailed review of previous LCA analysis was performed by Kargarzadeh et al. [115].

It is also important to note that eliminating or reducing the amount of chemicals will facilitate the utilization of a final stream of sugars for the production of other products, such as biofuels, aiding in the implementation of a biorefinery concept; differently, the utilization of other production routes may require expensive neutralization steps [10].

In the line of reduced utilization of chemicals, some benefits may also be found over the final nanomaterials. One of these could be an inferior potential toxicity towards the human body, facilitating their application throughout nanomedicine, cosmetics, food, and pharmaceutics [13]. It is worth noting that possible benefits of this aspect are essentially related to the final purification and preparation of the NC-based product, which will require a more/less complex process according to its manufacturing process. From the perspective of the purified material, either CNFs or CNCs, no significant differences in their cytotoxicity have been reported according to the production route. According to Xu et al. [116], different properties of a nanoparticle can dictate its cytotoxicity, such as its dimension, shape, reactivity, and other chemical or physical properties. Rather than the NC production method, these are more likely to affect the final cytotoxicity of the NC particles.

#### 5.6.2. High Selectivity and Improved Nanocellulose Properties

As a direct consequence of using enzymes, which can characteristically target more or less specific substrates or functional groups [117,118], the enzymatic NC production is commonly regarded as a highly selective and specific method [10]. Proper control of the enzyme dosage as well as the operational conditions and the composition of the enzymes cocktail, can allow a high process control, not only of the levels of degradation of the different solid components but also of the properties of the final material [103]. One of the main benefits from this control is the ability to prevent an extensive/thorough hydrolysis of cellulose [48,103], which would affect the extraction yield, but also the possibility to recover a clean sugar stream that can be easily used to produce value-added compounds [45]. The enzymatic extraction typically causes a decrease in the degree of polymerization and an increase of the crystallinity index, as a result of the hydrolysis of amorphous regions [119]. Another common advantage refers to a final NC product with a higher aspect ratio, as already observed by several authors [6,85,120].

A final aspect to also consider is that enzymatic extraction does not modify the surface chemistry of cellulose since no additional charges are introduced [103], usually resulting in a material with higher thermal stability [47,54,121]. As compared to sulfuric acid hydrolysis, this represents a clear advantage since the presence of sulfur groups leads to a negative surface charge [122].

#### 5.6.3. Economic Improvements

A most relevant aspect to consider on enzymatic NC production is the economics of the overall process, whether the enzymatic treatment represents a more economical option or is still not consensual. Adding to the economic benefits referred above and coming from an operation under mild conditions and a reduced chemicals utilization, the application of an enzymatic treatment will facilitate either the mechanical fibrillation or the production of CNCs, reducing the energy requirements to produce the same amount of NC material. While these benefits from enzymes are consensual, there is a clear lack of accurate estimations of the putative energetic savings introduced by enzymes. As pointed out by Rol et al. [51], this comparison should be carefully conducted since distinct extraction processes may produce NC particles with different modification rates and dimensions. A recent study by Espinosa et al. [25], employing an extrusion process after hydrolysis, reported a reduction of 37% of the energy consumption. In addition, Rol et al. [51] observed that the energy consumed to produce CNFs by extrusion (seven passes) decreased from over 15,000 kWh/t to approximately 5000 kWh/t when enzymes were employed; similar estimations of this type are, however, scarce.

On an opposite side, some authors have also pointed out that because of the cost of enzymes, which remains very high [123], this technology is still not very attractive from an economic point of view [13,22]. Nevertheless, as it may be possible to totally/partially reuse enzymes [58,124], it is conceivable to some extent that this cost could be tackled in the near future.

## 6. Nanocellulose Properties

### 6.1. Morphology and Size

NC morphology is strongly influenced by the production method, treatment conditions (e.g., treatment time, temperature, etc.), as well as by the source [125,126]. CNFs have a filament-like structure. They are long and flexible nanofibrils, consisting of both crystalline and amorphous domains. A CNF network can be seen as a nanoporous web-like structure of highly entangled nanofibers. On the other hand, CNCs are generally rigid needle- or rod-like nanoparticles obtained from crystalline parts of cellulose fibers [9,127]. Spherical CNCs have been reported in some works [48,64,76,86,128,129], and seem to be related to the higher severity of the process, such as high enzyme load.

Typically, CNFs present a high aspect ratio with a diameter less than 100 nm and a length of a few micrometers, depending on the production method [126,127]. Generally, CNCs diameter can vary from 10 to 50 nm and around 100 to 500 nm in length depending on the source [127]. Table 3 presents the sizes of CNFs and CNCs obtained from BEKP treated by enzymatic hydrolysis and other production methods, and as observed, CNCs from enzymatic hydrolysis have shown a greater length, as well as diameter, than those usually described for CNCs.

As previously referred, CNFs generally are produced by mechanical disintegration of the cellulosic fiber suspension under high shear forces, causing a longitudinal cleavage of the cellulose fibers into nanofibrils, while CNCs are usually produced by acid hydrolysis, typically with concentrated sulfuric acid under strictly controlled conditions, which after some kind of mechanical dispersion (i.e., sonication) yields nanocrystals. Currently, the enzymatic treatment has been used as an alternative or combined with a mechanical treatment aiming to decrease the energy spending of mechanical processes, which have high energy consumptions [127]. It has also been a greener alternative to acid hydrolysis since it operates under mild reaction conditions and without the generation of hazardous waste, as well as less water spent and processing steps. Furthermore, it allows recovering the hydrolysis products as a clean sugar stream, due to the specificity of the enzymes, which can be used to produce several value-added products [45,130]. In addition, the yields and morphologies of CNCs and CNFs can be tailored by controlling certain reaction conditions [11].

CNCs isolated by enzymatic hydrolysis have a higher aspect ratio (L/D) than that from acid hydrolysis [45,131]. Tibolla et al. [81] recovered CNFs with a higher aspect ratio (353.9 ± 28.2, being 7.6 ± 1.5 nm diameter and 2889.7 ± 214.3 nm length) from enzymatic treatment than from chemical treatment (42.7 ± 7.5, being 10.9 ± 2.3 nm diameter and 454.9 ± 6.6 nm length). On the other hand, Ma et al. [70] recovered CNCs from enzymatic hydrolysis with higher dimensions (28.4 ± 2.1 nm diameter and 343 ± 27 nm length) comparatively to acid hydrolysis (18.2 ± 1.9 nm diameter and 274 ± 32 nm length), but with a lower aspect ratio.

A previous step of enzymatic hydrolysis before the mechanical treatment has also shown to recover more uniform CNFs than only mechanical treatment, as reported by Zhu et al. [1], Qing et al. [36], Wang et al. [37], and Chen et al. [137]. Zhu et al. [1] verified that cellulase reduced the fiber length, resulting in CNFs with good uniformity in length. Wang et al. [37] also verified that a step of enzymatic hydrolysis previous to the mechanical fibrillation (microfluidization) allowed to obtain CNFs with the same length, but slightly thinner (lower diameter) and more uniform, while it also decreased the number of passes in the mechanical process, suggesting mechanical energy savings of at least 30% for nanofibrillation of cellulosic fibers.

### 6.2. Crystallinity and Polymerization Degree

During the process of NC production, the disordered amorphous regions of cellulose are removed resulting in the increase of the crystallinity [126,131], and depending on the treatment used or the suitable combination of them, NC with different degrees of crystallinity are obtained [34]. The length of the microfibrils is related to the degree of polymerization (DP) of constitutive cellulosic chains or molecular weight, and depending on the enzyme hydrolysis process before fibrillation, cellulosic chains with different DPs can also be obtained [131].

Zhu et al. [1] followed the crystallinity and polymerization degree of cellulose pulp during 72 h enzymatic hydrolysis and verified that crystallinity index (CI) increased from 46% to 57% (an increase of 24%) after 48 h of hydrolysis. Significant reductions in DP throughout the hydrolysis process were also observed due to the action of cellulases, that not only hydrolyzed the cellulose chain but also significantly reduced the fiber length (from 1.2 mm to 200 μm) after 24 h hydrolysis, resulting in a substrate with good uniformity in length. A decrease in the DP was also observed by Tarrés and co-authors [39] by increasing the enzyme load and the hydrolysis time.

The crystallinity study of the produced NC is important to understand the effect of production methods on the crystal structure of NC. In addition, by increasing the severity of the hydrolysis (increasing the hydrolysis time or enzyme load), it is possible to depolymerize the crystalline cellulose, decreasing the length of the particles, and their aspect ratio, and eventually resulting in spherical particles [138]. Tong et al. [48] and Chen et al. [64] reported the production of spherical CNCs by increasing the enzyme load. De Aguiar et al. [11] studied the production of CNCs from sugarcane bagasse and straw and verified an increase in the CI of CNCs obtained after 24 h of enzymatic hydrolysis, indicating that enzymes acted on the removal of amorphous regions of cellulose. However, as hydrolysis time increased, there was a slight decrease of CI, which could indicate that the enzymes started to depolymerize the crystalline regions of cellulose. The increase of the hydrolysis severity also led to lower CNC dimensions (length and diameter). Cui et al. [47] verified a decrease in CI at the highest severity condition that corresponded to 120 h hydrolysis and 60 min ultrasonic treatment.

### 6.3. Mechanical Properties

CNFs present a dense network held together by strong interfibrillar bonds, resulting in enhanced mechanical properties. This is related to the relatively high crystallinity of NC, the abundance of hydroxyl groups on their surface, and the high aspect ratio that promotes this reinforced structure [4,127]. Crystallinity and polymerization degree influence the physical properties of nanofibrillated cellulose matrix, wherein a high DP of cellulose leads to films with higher tensile strength [9,16,34]. The aspect ratio presents an important role in determining the nanofiber’s reinforcing capacity, as it affects the fiber’s ability to maintain the film network [53]. Moreover, the high surface area of the CNC also induces great mechanical properties [127].

CNFs and CNCs have a higher axial elastic modulus and tensile strength compared to the synthetic fiber Kevlar that is used commercially to reinforce plastics. In general, their mechanical properties are within the range of materials used as reinforcement, such as carbon fiber. These characteristics are attributed to their crystalline regions, appearing as a bundle of stretched cellulose chain, with axial elastic modulus values ranging between 110 and 220, tensile strength up to 7.7 GPa, and a density of 1.6 g/cm^3^ [9,34,138].

Several works have investigated the mechanical properties of NC composites. Xu et al. [135] compared the incorporation of CNCs and CNFs on polyethylene oxide (PEO) films and verified that, for the same NC concentration, CNFs led to higher strength and modulus than CNCs due to the higher aspect ratio of CNFs and to the entanglement of fibers, but with less strain-at-failure due to their relatively large fiber clusters. Tibolla et al. [57] prepared nanocomposite films with CNFs from enzymatic hydrolysis, verifying that these had lower elongation at break and higher Young’s modulus and tensile strength than the control film. The most affected property by CNFs was Young’s modulus, due to an increase in more than 100% with the incorporation of CNFs. In addition, the films reinforced with CNFs1 (L/D = 404.5) were more rigid and brittle than that with CNFs2 (L/D = 170.2), suggesting that the aspect ratio of the nanofibers might be a parameter influencing the mechanical properties of films. CNFs1 presented lower length and diameter (1490 ± 107.3 nm and 3.7 ± 0.4 nm) than CNFs2 (1544.5 ± 40.6 nm and 8.8 ± 0.7 nm); thus, CNFs number per unit area in the films could explain the arrangement of a more homogeneous, interconnected, and rigid structure with a reduction of CNFs1 film elongation, suggesting that mechanical properties of films seem to be affected by the size of CNFs.

Zhao and co-authors [139] verified that by varying the content of CNFs on all cellulose nanocomposite films, Young’s modulus of the nanocomposite films increased from 0.76 GPa to 4.16 GPa and the tensile strength from 61.56 MPa to 99.92 MPa. Zhu et al. [1] produced films using CNFs obtained through enzymatic hydrolysis + microfluidization and verified that the tensile strength (maximum stress) of the CNF film was 45 ± 5 MPa, or more than an order of magnitude greater than sheets made with the control fibers (3.7 ± 0.3 MPa). The modulus of the CNF film was 5400 ± 180 MPa, or about six times of the sheet made from the control fibers (900 ± 60 MPa). Control films corresponded to pure hydrolyzed fibers with no microfluidization, showing that the mechanical treatment after the enzymatic hydrolysis was important to enhance the mechanical properties of films.

On the other hand, Zhao et al. [74] compared the mechanical properties of softwood CNFs from TEMPO-mediated oxidation (tSW) and enzymatic hydrolysis (eSW), as well as the hardwood CNFs from enzymatic hydrolysis (eHW), verifying that the tensile stress of tSW and eSW was similar (81.8 and 79.0 MPa), while the modulus was significantly higher for tSW (10.6 GPa) comparatively to eSW (5.8 GPa). The authors attributed this result to the introduction of anionic charged carboxyl groups that could induce the formation of additional hydrogen bonds to those from the original hydroxyl groups, hence contributing to more inter-fiber bonding and thus a stronger network. Another possible explanation suggested by the authors was that the tCNFs are more evenly distributed prior to the film formation, and thus fewer “weak points” are formed. Moreover, films from softwood (eSW) were slightly stronger than those from hardwood (eHW), as seen by the tensile stress of 79.0 vs. 69.0 MPa and Young’s modulus of 5.8 GPa vs. 4.6 GPa. The strength of a film showed to be closely correlated to the DP, crystallinity, and aspect ratio of the CNFs used. In addition, Tarrés et al. [40] compared the mechanical properties of nanopapers produced from TEMPO-mediated oxidation CNFs (CNFs-T) and enzymatic CNFs (CNFs-E) and verified that CNFs-E were weaker at tensile than CNFs-T, but with similar stiffness levels. According to the authors, the strength differences of nanopapers from CNFs-T and CNFs-E are probably due to the differences in the intrinsic properties of each CNF, such as higher specific surface and fibrillation yield for CNFs-T, a condition that gives CNFs-T a greater swelling capacity and, therefore, better ability to form a three-dimension network.

### 6.4. Thermal Properties

Thermal stability is an important property for NC since the processing temperatures of most composite materials are usually above 200 °C [43,54]. Cellulosic materials degrade below 400 °C (generally decomposition starts at 310 °C and persists until 400 °C) [53], with the degradation temperature dependent on the structure and chemical composition [126].

It has been shown that the particle size and the pretreatment used to obtain the cellulosic material affect the thermal properties. As a result, CNFs start to decompose at a higher temperature (350 °C) than CNCs (thermal degradation starts typically between 200 and 300 °C) [28,125]. Reduced thermal stability of CNCs is related to sulfuric acid hydrolysis that results in CNCs with high colloidal stability, due to the introduction of negatively-charged sulfate groups, but also in a significantly lower thermal stability, which limits several applications requiring high temperatures for processing [3,43,45]. The sulfate groups degrade around 120 °C and they decrease the thermal stability of cellulose [126]. CNCs with lower sulfate content have better thermal stability [125], which can also be increased by neutralizing the sulfate groups [140].

On the other hand, CNCs produced by enzymatic hydrolysis present a high number of free hydroxyls on their surface that can interact strongly among each other through hydrogen bonds and van der Waals forces, which can confer low colloidal stability regarding CNCs produced by sulfuric acid, but higher thermal stability [45,121,130]. Therefore, enzymatic hydrolysis offers an attractive way to produce NCs with superior thermal stability, contributing to further expansion of their range of applications [43,141].

Squinca et al. [43] observed that CNCs produced from enzymatic treatment presented higher thermal stability regarding the mechanical one, attributing this finding to the increased crystallinity. In addition, De Aguiar et al. [11] verified an increase of the thermal stability of CNCs produced by enzymatic hydrolysis (Tonset at ~310 °C) when compared to the crude and treated fibers. However, by increasing the hydrolysis time, the thermal stability of the fibers decreased, which may be related to a reduction in the size of the nanoparticle, which was observed with the extended time of hydrolysis, or to the decrease in CI as well. On the other hand, Chen et al. [64] produced rod-like and spherical CNCs, according to the enzyme load, and reported similar thermal stability (Tonset at ~205 °C and Tmax at ~345 °C). Furthermore, Bondancia and co-authors [54] reported that CNFs and CNCs obtained by enzymatic hydrolysis presented superior thermal stability compared to materials obtained by means of chemical hydrolysis reactions. They reported degradation of cellulose pulp at 331 °C, CNFs (24 h) at 323 °C, and CNCs (144 h) at 325 °C. Lastly, Zhao et al. [74] observed that CNFs from enzymatic hydrolysis had higher thermal stability than CNFs from TEMPO-mediated oxidation.

### 6.5. Barrier Properties

Nanocellulose research involving barrier properties has mainly focused on water vapor and oxygen permeabilities. Cellulose is a hydrophilic polymer, absorbing water when subjected to a moisture atmosphere, however, a decrease in water vapor permeability (WVP) has been seen for nanoscale cellulose [4,142]. The barrier properties of nanocellulose are related to its relatively high crystallinity, associated with its capacity to form a dense network held together by strong interfibrillar bonds, which make diffusion of molecules in the crystalline domains of cellulose fibrils difficult [4,143].

The barrier properties have been studied mainly for MFC films that have shown outstanding performance. Belbekhouche et al. [143] studied the influence of the nature of the nanoparticles (CNC and MFC) on the barrier properties of the films, that is, WVP and gas (carbon dioxide, nitrogen, and oxygen) permeability. Surprisingly, CNC films presented higher water vapor sorption than MFC films. Once CNC presented a more organized structure and highly crystalline structure than MFC, the authors attributed this result to the presence of residual lignin, extractives, and fatty acids at the surface of MFCs, which could result in MFC films with higher hydrophobicity. CNC films also presented higher permeability to gases than MFCs, showing that other factors, such as the highest porosity of the CNC films and the MFC’s entangled structures that lead to an increase in the tortuosity of the diffusion path, may have a greater influence on the barrier properties than the crystallinity.

Other works have related the dependence of barrier properties of MFCs, mainly WVP, with different factors, such as MFC source, production method, and chemical modification of MFCs [16]. Spence et al. [144] produced MFCs from different wood pulp sources (bleached softwood and hardwood, and unbleached ones containing or not lignin), and compared the water vapor transmission rate (WVTR) of MFC films with the source and chemical composition. They compared the WVTR of the original pulps with those of the corresponding MFCs and verified that the processing to convert the macrofibrils into MFCs resulted in a decrease in the WVTR (from 20% to 30%). MFCs from bleached hardwood presented the highest water vapor barrier (200 g/m^2^/day) among the different sources, and the lignin-containing sources presented higher WVTR, which may be due to the larger pores in the films (from 300 g/m^2^/day to 460 g/m^2^/day). Thus, the chemical composition of wood pulps appears to have a greater effect on water barrier properties than the type. Minelli et al. [142] prepared MFC-based films using two kinds of MFCs, with similar source and composition, but with two different processing methods, that is, enzymatic treatment (MFC G1) and carboxymethylation treatment (MFC G2); both were followed by high-pressure homogenization, resulting in MFCs with different dimensions (17–30 nm diameter MFC G1 and 5–15 nm MFC G1) and anionic charge densities (~40 mequiv./g for MFC G1 and ~586 mequiv./g for MFC G2). Carboxymethylated MFC films presented a more compacted nanofibril network (as reveled by FE-SEM images) and lower WVP than the enzymatic MFC films, however, the differences were small, suggesting that the treatment did not play an important role in water vapor properties.

Moreover, the physical structure of the MFCs also affects the WVP of the films [16]. Tibolla et al. [57] incorporated CNFs (also designated MFCs) isolated through enzymatic hydrolysis on starch-based films and verified that water barrier properties were dependent on the CNFs size and aspect ratio. Films produced with CNF1 showed a reduction in WVP, while CNF2-based films (higher diameter and length size, and then lower aspect ratio than CNF1) showed an increase. Longer CNFs tend to agglomerate, facilitating the water molecules’ permeation through the polymer matrix gaps, while smaller CNFs are more properly dispersed into the matrix, showing that the poor performance of nanocellulose composites is also related to the poor dispersion of the filler.

Nanocellulosic materials have been reported to present good oxygen barrier properties. Syverud and Stenius [145] reported a low oxygen transmission rate (OTR) for MFC films (17–18 mL/m^2^/day at 0% RH (Relative humidity) for films 21–30 μm thick), that was comparable to the synthetic PVdC-coated, oriented polyester (9–15 mL/m^2^/day), with similar thickness. The recommended OTR for modified atmosphere packaging is less than 10–20 mL/m^2^/day [146]. Fukuzumi et al. [147] reported a significant decrease in oxygen permeability at 0% RH of 25 μm tick PLA (polylactic acid) film (746 mL/m^2^/day/Pa), when it was coated by a thin (0.4 μm) TEMPO-oxidized cellulose nanofiber (TOCN) layer (1 mL/m^2^/day/Pa), making it, according to the authors, comparable to oxygen permeability of typical synthetic polymers that have high oxygen barrier property, such as poly(vinylidene chloride) and polyethylene-poly(vinyl alcohol) copolymers. Similar behavior was observed by Rodionova et al. [148] for PET (polyethylene terephthalate) films coated by TOCN.

The low permeability to oxygen of the nanocellulose, in dry conditions (0% RH), is related to its high crystallinity and dense structure. However, its performance is strongly limited by its hygroscopic properties and consequent moisture sorption, which creates less packed films, reducing the barrier with higher relative humidity, as verified by Minelli et al. [142], who reported that MFC films also showed excellent oxygen barrier properties in dry conditions. However, in wet conditions, MFC films showed an increase in permeability, as observed for hydrophilic molecules. Chemical modification on MFC (i.e., carboxymethylation) was developed by Aulin et al. [149] to improve the oxygen barrier properties of nanocellulose films at high RH level.

### 6.6. Optical Properties

The influence of cellulose structures on optical properties of composites is related to different characteristics such as size, shape, ordering, charge, and dispersity of the cellulosic material into the matrix [150]. Additionally, NC concentration in the composite and its thickness can affect the transmittance and clarity of the final material [34].

CNFs possess great optical properties, considering the size of the CNFs is less than the wavelength of visible light [126,151]. Films prepared with CNFs appear smoother and less porous than the traditional cellulosic papers and have a certain level of transparency (light transmission) depending on the CNF dimensions. On the other hand, CNCs can organize themselves in a chiral nematic way in suspensions leading to the formation of iridescent films once dried, unlike CNFs [152]. However, even though the films produced by CNCs are transparent, they are usually more brittle than those of CNFs due to their crystalline nature [127].

Xu and co-authors [135] showed that PEO/CNC films exhibit higher transparency than PEO/CNF films, most likely due to CNCs’ smaller sizes and lack of agglomeration and entanglement. Tarrés et al. [39] observed that the transmittance of the CNFs in general increased with the increase of the enzyme load and hydrolysis time, which are related to the higher yields of nanofibrillation. Furthermore, Zhu et al. [1] produced films using CNFs obtained through enzymatic hydrolysis + microfluidization and verified that the opacity of the films clearly decreased with the number of passes through the microfluidizer, where films produced from highly nanofibrillated cellulose (60 passes) had opacity values of only approximately 12%. On the other hand, CNFs produced from TEMPO-mediated oxidation showed much higher transmittance than those from enzymatic hydrolysis [38,40,74]. According to the authors, this could be related to the introduction of surface charge on TEMPO-CNFs, which improves cellulose disintegration resulting in more elementary fibrils, resulting in films more uniform and of higher density, as well as with a higher diameter and lower fibrillation yield of enzymatic CNFs.

### 6.7. Rheological Properties

As a final point, rheological studies can give information about the fibrillation state of the particles [131]. Some cellulose-based structures present interesting rheological properties that can be described in terms of pseudo-plasticity and shear-thinning behavior [34]. The particularity of NC is that their dispersions present unusual rheological properties [153]. These NC particles are able to immobilize a high amount of water into developed external and internal surfaces with the production of highly viscous gel-like water systems [4].

Pääkkö et al. [63] studied the rheological behavior of enzymatically treated MFC and the relation between this behavior and the mechanical properties of these materials. The study showed that MFC suspensions displayed a gel-like behavior from concentrations of 0.125% to 5.9%. The values of the storage modulus (G′) were rather high; about 10^4^ Pa was reported for a 3% MFC suspension, as compared to 10^2^ Pa reported previously by Tatsumi et al. [154] for a 3% suspension of rod-like CNCs. In addition, the storage modulus was particularly dependent on the MFCs’ concentration, where increasing MFCs’ concentration from 0.125% to 5.9% resulted in an increase in the storage modulus by five orders of magnitude. The authors attribute this high elastic modulus to the long fibrils and fibrils aggregates, which form an inherently entangled network structure.

On the other hand, Tang et al. [65] studied the rheological behavior of CNC suspensions obtained from acid hydrolysis/sonication process, acid hydrolysis/enzymatic hydrolysis (1 h)/sonication process, and acid hydrolysis/enzymatic hydrolysis (24 h)/sonication process. All CNC suspensions exhibited a visible shear-thinning behavior; however, the CNCs from enzymatic hydrolysis treatment presented a strong reduction in viscosity, which may be related to the progressive removal of amorphous regions of cellulose, leading to a reduction in DP. In addition, as the angular frequency increased, the storage modulus of CNC suspensions increased, although this increase was less evident with the increase in enzymatic hydrolysis time, indicating a gel-like behavior due to the high aspect ratio of the nanofibril gelation network, as suggested by the authors.

## 7. Market and Applications

The nanoscale dimensions, high aspect ratio, and outstanding reinforcing potential of NC have increased the demand for NC to be used in nanocomposites. The market for NC is expected to grow from 297 million USD in 2020 to 783 million USD by 2025, at a compound annual growth rate (CAGR) of 21.3%. Pulp and paper represent the majority of the overall market, being used as additives in papermaking to produce lighter and stronger paper and board providing improved properties, such as less porosity, higher printing quality, and less transparency [155].

**Figure 4:**
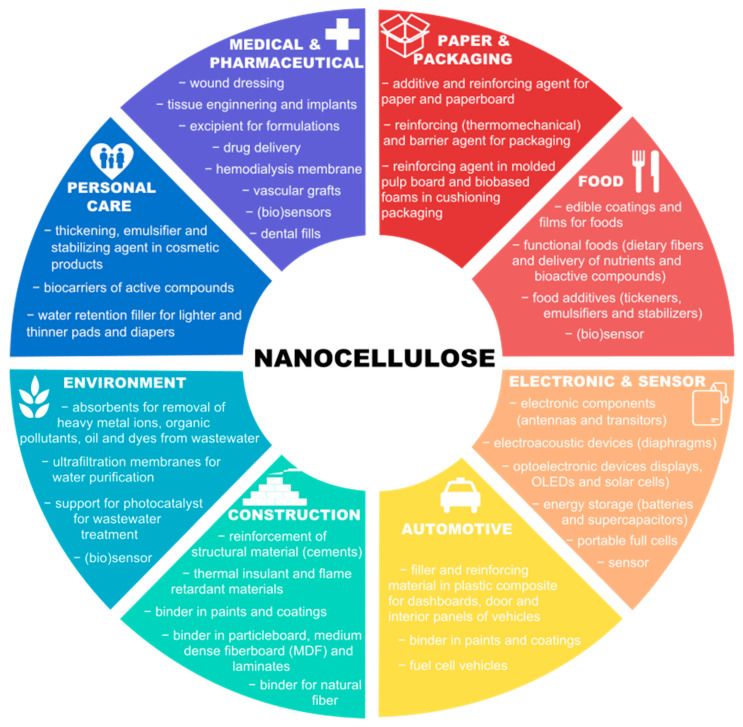
The potential application of nanocellulose in different fields.

Recently, NC has attracted a lot of attention as a potential filler in nanocomposites (Figure 4). It has been reported that the incorporation of NC in a wide range of polymer matrices improves their mechanical properties. Table 4 reports the applications of NC produced by enzymatic hydrolysis in different fields. In the food industry, NC has been incorporated in a polymer matrix to improve the mechanical properties of the nanocomposite to be used as coating and films in food packaging, providing protection to ensure food safety and quality, and shelf life of perishable foods. NC films also show excellent oxygen barrier properties related to the high crystallinity, a network structure held together through strong inter- and intramolecular hydrogen bonds, a lamellar nanofiber structure, and dense fiber bundles [156,157,158]. CNFs have also been used as filler in all-cellulose nanocomposite films [139,159]. In addition, NC, as a dietary fiber, can be used in functional foods to reduce the risk of chronic diseases such as diabetes, obesity, cardiovascular disease, and diverticulitis. It also fosters beneficial physiological effects, such as laxation and attenuation of blood cholesterol and glucose and has been explored to produce reduced-fat foods to treat weight disorders. NC based-hydrogels have also been applied for delivery of nutrients or bioactive compounds, protecting the stored compound and controlling their release into the gastrointestinal system [160]. NC can also be used as thickeners and suspension emulsifiers and stabilizers in a wide variety of food products (food additives) [156,157,158]. Recently, VTT produced cellulose-based materials from enzymatic fibrillated cellulose (HefCel) technology. These HefCel CNFs can be used as reinforcement of paper and packaging materials [161].

NC has shown great potential in electronic applications. NC has been used as a flexible and transparent substrate, such as paper and polymer films, for the development of flexible electronic devices, such as solar cells, sensors, and flexible displays. CNFs have been the most used substrate in electronic devices rather than CNCs probably due to the more brittle nature of CNCs than CNF films, which makes them more suitable for flexible substrates. NC is also a promising bio-based material in the synthesis of conductive materials as it can act as (i) a bio-template for the synthesis of tubular conductive particles using NC with a high aspect ratio; (ii) a capping and nucleating agent in the synthesis of metallic particles; and (iii) a dispersing and stabilizing agent or (iv) a binding agent in ink formulation. It has been used for the fabrication of energy devices, such as batteries and supercapacitors [127,162]. Vilela et al. [163] revised the application of NC in polymer electrolyte fuel cells, namely the ion-exchange membrane and electrocatalyst, as well as in the application of NC-based components in microbial fuel cells (MFCs).

NC has also been applied in environmental remediation as a new generation of nanostructured adsorbents for different classes of pollutants due to their large surface area, vast surface hydroxyl groups, and their easy functionalization [111,164]. In general, these NC-based adsorbents need to pass by a surface functionalization process to be able to enhance their adsorption capacity and adsorb a specific class of pollutant [165]. NC-based adsorbents, such as aerogels, filtration membranes, porous beads, and flocculants, have been used for removal of heavy metal ions (Cu^2+^, Pb^2+^, Hg^2+^), organic pollutants (pesticides such as DTT (dichlorodiphenyltrichloroethane), industrial chemicals such as PCBs (Polychlorinated biphenyls), and substances such as dioxins), dyes (methylene blue, Congo Red, rhodamine) and oils in water purification systems and wastewater treatment, and for removal of air contaminants.

In the biomedical field, CNC and CNF hydrogels have been used for many biomedical applications, such as wound dressing, drug delivery, and tissue engineering, due to their biocompatibility, biodegradability, mechanical properties, and the advantages of a nanostructure. In addition, NC has antimicrobial activity, wound healing properties, a non-toxic nature [110,166], and also a high binding potential through the available OHs and negative interfacial charges, which make their electrostatic adsorption on tissues easier [167].

In wound dressing, NC provides a suitable environment for the wound healing process for burnings and wounds: it provides an infection barrier; absorbs the purulent fluids in exudative wounds; allows good oxygen permeability for cellular proliferation and to prevent the growth of anaerobic bacteria; keeps the wound surface moist; has a painkiller effect; and has a high degree of adherence to various types of tissues [125,160]. In addition, the translucency of CNFs helps to follow the development of the wound without the need to remove the dressing [168]. In drug delivery systems, it allows a controlled and sustained release profile. Drug carriers based on NC are generally classified in microparticles, hydrogels, gels, membranes, and films [110]. In recent years, stimuli-responsive CNF-based hydrogels received much attention, due to the drug-releasing performance under specific stimulus control, for example, pH, temperature, and ionic strength [166]. CNC- and CNF-based hydrogels have been broadly used in tissue engineering in the last years due to their highly hydrated three-dimensional porous structure that mimics biological tissue, as well as their outstanding mechanical property [166,169]. Thus, much attention has been paid to the 3D printing technique for fabricating implants and scaffolds for tissue engineering applications [111,166,170]. Table 4 reports the application of NC produced by enzymatic hydrolysis in different fields.

## 8. Final Remarks

The cost involving NC production still makes large-scale operations difficult. In recent years, the increasing search for environmentally friendly and biodegradable materials has driven the development of processes using enzymatic hydrolysis. The NC production by enzymatic treatment presents a viable alternative to scale-up the NC production, since it may contribute to decreasing the total cost of the process, as well as the environmental impact related to others production methods. NC production by enzymatic treatment presents several advantages, as discussed previously, namely mild reaction conditions, no production of hazardous waste, reduced consumption of water, and the possibility of using the secondary stream of fermentable sugars released in the hydrolysis to obtain other bio-based products. Moreover, NC production by enzymatic hydrolysis has been reported to result in easily functionalized nanostructures, with high thermal stability, specific surface area, and aspect ratios. Currently, enzymatic hydrolysis has been explored to decrease the energy requirement to obtain CNFs via mechanical treatment or eliminate/minimize the use of hazardous chemicals, such as sulfuric acid and TEMPO. However, the cost of enzymes is one of the main factors that determine the economics of the production process.

The development of enzymes at a competitive cost of production is a challenge, and many efforts have been made in this regard by many research groups. It is estimated that the culture medium represents more than 50% of the total costs of enzyme production, with the carbon source as the most expensive component. Therefore, significant efforts have been made to genetically modify *T. reesei* in order to diversify the carbon sources that it can utilize for enzyme production. Recently, a research group reported the development of an industrially relevant cellulase production platform, using *T. reesei* RUT-C30 strain. In this work the implementation of some genetic modifications and the development of a process based on sugarcane molasses, a low cost and highly concentrated sugar-rich product that contains many vitamins and minerals, allowed to achieve 80.6 g/L of cellulase, being the highest levels of cellulase reported experimentally to date for *T. reesei*. Moreover, the saccharification efficiency of the enzyme cocktail produced was similar to that of a cellulase preparation available on the market [177]. Thus, studies point to the economic viability of producing cellulases in the near future, and consequently the NC production by enzymatic hydrolysis.

## Figures and Tables

**Table 1:** Nanocellulose production, method of production, and yield obtained per 100 kg of raw material.

Source of Cellulose	Production Method	Chemical/Enzymatic Load *	NC Type	Yield (kg)	Reference
Sugarcane bagasse (SB) Sugarcane straw (SS)	Alkali treatment	2000 kg NaOH;	CNC	11.3 (SB) 12 (SS)	[11]
	Bleaching	680 kg NaOH; 190 kg Acetic acid; 42 kg NaClO_2_			
	Enzymatic treatment	3.2–4.6 kg protein (Cellic CTec3)			
Wheat straw	Soda pulping	7 kg NaOH	CNF	42.3	[25]
	Enzymatic treatment	18,600 ECU FiberCare^®^ endoglucanase			
Banana peels	Delignification treatment	100 kg KOH; 20 kg NaClO_2_	CNF	27–71 **	[12]
	Acid treatment	2–200 kg H_2_SO_4_			
Wood flour	Alkali treatment	100 kg calcium hypochlorite; 10 kg acetic acid	nano structures	11.43	[41]
	Alkali bleaching	40 kg NaOH; 240 kg H_2_O_2_			
	Enzymatic treatment	5.4 kg Cellic CTec2; 0.6 kg Cellic HTec2			
Grapevine stems	Alkaline treatment	45 kg NaOH/100 kg r.m.	CNF	15–20	[6]
	Bleaching	18 kg NaClO_2_/100 kg alkali-treated r.m.			
	TEMPO Oxidation	1.6 kg TEMPO radical; 26 kg NaBr; 900 kg NaClO/100 kg of cellulose			
Grapevine pomace	Alkaline treatment	45 kg NaOH/100 kg r.m.	CNC	10–15	[6]
	Bleaching	18 kg NaClO_2_/100 kg alkali-treated r.m.			
	Acid Hydrolysis	500 kg H_2_SO_4_/100 kg cellulose			
Cotton linters	Enzymatic treatment	200–2000 U of cellulose C on an Ahiba Easydye	CNC	80	[42]

* calculated from published data; ** per 100 kg of cellulose; ECU: endocellulase units; r.m: raw material.

**Table 2:** General description of previous studies reporting the production of CNFs and CNCs by enzymatic hydrolysis.

NC Type	Raw-Material	Before EH	After EH	Enzyme	Enzyme Dosage	Operational Conditions	Dimensions/ Crystallinity	Reference
CNF	Wheat straw	Alkaline treatment	Twin-screw extrusion	FiberCare^®^	300 ECU/g	2% solids; pH 5; 50 °C; 2 h	D: 15 nm L: 991 nm CI: 58%	[25]
	Lemongrass leaves	Steam explosion; delignification	Sonication	Viscozyme^®^ L	--	0.5% solids; pH 4.8; 50 °C; 24 h	D_h_: 106 nm CI: 49%	[56]
	Banana peel	--	--	Xylanase (Novozymes)	70 U/g	15/35% solids; pH 6; 35/55 °C; 24 h	D: 3.7–8.8 nm L: 1490–1545 nm CI: 61.5–66.2%	[57]
	Bagasse pulp	Sonication	--	Cellulase (Sigma)	10 discs of immobilized enzyme	0.4% solids; pH 5; 50 °C; 6 h	D: 15–35 nm	[58]
	Citrus pulp from oranges	--	Chemical treatments; Sonication	*Xanthomonas axonopodis* lysate	5 mg protein extract/g solid	17% solids; pH 4.8; 45 °C; 24 h	CI: 60%	[59]
	Soybean straw	Chemical treatments	Homogenization; Sonication	Optimash™ VR	134 U/g solid (Endoglucanase)	2% solids; pH 4; 50 °C; 42 h	D: 9.4 nm CI: 50%	[60]
	Bleached eucalyptus Kraft pulp	--	Extrusion or Grinding	FiberCare^®^ R	300 ECU/g cellulose	2% solids; pH 5; 50°C; 2 h	D: 25.8 nm CI: 70–80%	[51]
	Orange peel	--	Grinding	Pectinase Amano PL™	1 or 10 mg protein/g solid	5% solids; 45 °C; 24 h	D: 10–50 nm CI: 59.2%	[61]
	Curauá fibers	Chemical treatments	Sonication	FiberCare^®^ R and Viscozyme^®^ L	ranging levels	2% solids; pH 4.8; 50 °C; 72 h	D: 55–109 nm L: 1280–4100 nm CI: 73–78%	[62]
	Bleached sulfite softwood pulp	--	Homogenization	Novozym 476	0.85 ECU/g fiber	4% solids; pH 7; 50 °C; 2h	D: 5–6 nm	[63]
CNC	Sugarcane straw	Chemical treatments	--	Cellic^®^ CTec3	10 mg protein/g fiber	10% solids; pH 5; 50 °C; 96 h	D: 8.7–14.1 nm L: 395.5–507.7 nm CI: 66.7–70.4%	[11]
	Eucalyptus cellulose Kraft pulp	Ball milling	Sonication	On-site production by *A. niger* strain	20 mg protein/g solid	2% solids; pH 4.8; 50 °C; 48–96 h	D: 24 nm L: 294 nm CI: 77.9–78.3%	[43]
	Cotton pulp	Swelling treatment	Sonication	Cellulase (Ningxia Xiasheng)	10–300 μ/mL	1% solids; 50 °C; 5–11 h	D: 30–45 nm L: 250–900 nm	[64]
	Bleached eucalyptus Kraft pulp	--	Sonication	Monocomponent EGs	400 U/g pulp	2% solids; pH 4.5–6; 50 °C; 72 h	D: 6–10 nm L: 400–600 nm CI~88%	[45]
	Cotton linters	--	Acid hydrolysis; Sonication	Cellulase preparation (Fungal Bioproducts)	2–20 U/g pulp	5% solids; pH 5; 55 °C; 2–24 h	Z average: 183–209 nm	[42]
	Sugarcane bagasse	Steam explosion/Liquid hot water	Chemical treatment; acid hydrolysis	Cellic^®^ CTec2	7–22 mg protein/g cellulose	10% solids; pH 5; 50 °C; 24 h	D: 14–18 nm L: 195–250 nm CI: 77.7–81.7%	[2]
	Wheat microcrystalline cellulose	Sonication	--	Celluclast 1.5 L	0.5 mL/g solid	3% solids; pH 4.8; 50 °C; 72–120 h	D: < 10 nm L: 40–200 nm CI: 74.4–87.5%	[47]
	Old corrugated container	Chemical treatments	Sonication	Celluclast 1.5 L	1 mL/g fiber	0.8% solids; 50 °C; 1–36 h	D: 15–80 nm L: 100–400 nm CI: 57.8%	[65]
	Northern bleached hardwood Kraft pulp	--	Acid hydrolysis; dialysis	Proprietary EG preparations	0.2–5 U/g pulp	10% solids; pH 4/7; 50 °C; 72 h	D_h_: 125–148 nm CI: 77–83%	[66]

EH—Enzymatic hydrolysis; D—diameter; L—length; D_h_—hydrodynamic diameter, CI—crystallinity index.

**Table 3:** Size of NC obtained from bleached eucalyptus Kraft pulp (BEKP) from enzymatic treatment and other production methods.

NC Type	Production Method	Process Description	Diameter (nm)	Length (nm)	Reference
CNC	Enzymatic	2% solids, 20 g protein/g solid of enzyme loading, 50 °C, 96 h reaction + sonication	24 ± 4.3	294 ± 66.8	[43]
		2% solids, 400 U/g of pulp of endoglucanase loading, 50 °C, 72 h + sonication	6–10	400–600	[45]
		10% solids, 1–50 U/mL enzyme loading, 50 °C, 12 h	20–40	600–800	[48]
		CNF extended hydrolysis using 20% solids, 10 mg protein/g of enzyme loading, 35 °C, 144 h	15 ± 6	216 ± 86	[54]
	Acid Hydrolysis	58% sulfuric acid, 56 °C, 40 min or 62% sulfuric acid 62%, 50 °C, 70 min + ultrasound bath treatment	10.7 ± 8.5 17.3 ± 6.1	174 ± 125 204 ± 129	[132]
		63.8% sulfuric acid, 45 °C, 1 h	8	125	[133]
		60% sulfuric acid 45 °C, 30 and 60 min	15 ± 6 11 ± 4	175 ± 38 142 ± 49	[134]
		64% sulfuric acid, 45 °C, 25 min + sonication	4.8 ± 0.4	147 ± 7	[122]
		Sulfuric acid	19 ± 5	151 ± 39	[135]
		mixture (2:1 *v*/*v*) 60% sulfuric acid and 36.5% hydrochloric acid, 45 °C, 75 min under mechanical stirring	23 ± 5	200 ± 50	[136]
CNF	Enzymatic	20% solids, 10 mg protein/g of enzyme loading, 50 °C for 24 h	21± 3		[54]
		10% solids, 5 and 10 mg protein/g cellulose, and 15% solids, 10 mg protein/g cellulose, 50 °C, 24 and 48 h	18–31		[55]
		5% refined pulp, 0.24 g of enzyme Novozyme 476/kg of dried fibers, 50 °C, 4 h	23.8		[38]
	Enzymatic Mechanical	10% (*w*/*v*) solid, 5–10 FPU/g substrate, 50 °C, 48 h + mechanical homogenization (microfluidizer 60 passes)	20	500	[1]
		10 g pulp, 0.4 g cellulase, 50 °C, 10 h + Mechanical grinding at 1500 rpm twice	69.1 ± 15.2	2378 ± 940	[137]
		2% refined cellulose pulp, 300 U endoglucanase/g of cellulose, 50 °C, 2 h + twin screw extruder (TSE) at 400 rpm (1–7 passes)	25.8 ± 7.1		[51]
		Enzymatic (E): 10% solids, 3 FPU/g fiber, 50 °C, 24 h + refining (R): mechanical fibrillation 1500 rpm, 6 h + microfluidization (15 times)	38 ± 21 (ER) 12 ± 2.4 (ERM)		[36]
		5% solids, 1 mg protein (Ph-GH5)/g fiber, 70 °C, 48 h + microfluidization (30 passes)	5–10	1–2 um	[37]
		5% refined fibers, 80–320 g Novozym 476/Tn, 50 °C, 4 h + homogenization (total 9 passes)	37.7–25.1	1009–559	[40]
	Mechanical	Refining (R): intact pulp was refined until refining level of CSF 100 mL (several times) or Sonication (S): milled pulp was sonicated for 7 h (with 30 min interval)	<100 (R) 20–50 (S)	<500 200–2.5 mm	[134]
		Multi-pass high pressure grinding process	20 ± 14	1030 ± 334	[135]
		Grinding (30 passes)	17 ± 4	1.1 ± 0.5 um	[136]
		Grinding at 1500 rpm twice	118.6 ± 62.6		[137]
		Refining (R): mechanical fibrillation 1500 rpm, 6 h + microfluidization (15 times)	15 ± 6.2		[36]
		Microfluidization (40 passes)	5–14	1–2 um	[37]

**Table 4:** Application of nanocellulose in different fields.

NC Type	Production Method	Composites	Application	Description	Reference
MFC	Mechano-enzymatic	MFC/ lignosulphonate (LS)	Carbon precursors	MFC/LS hydrogels were used in the manufacture of carbon objects by 3D printing and carbonization	[171]
CNF	Mechanical refinement and enzymatic treatment	CNFs/alginate	Cartilage tissue engineering	CNF/alginate bioink was used for 3D bioprint of human-derived induced pluripotent stem cells (iPSCs) into cartilage mimics in co-cultures with irradiated chondrocytes	[172]
CNF	Enzymatic hydrolysis with mechanical shearing and high-pressure homogenization	CNFs/alginate	Cartilage tissue engineering	CNF/alginate bioink was used for 3D bioprint human chondrocytes.	[173]
CNF	Mechano-enzymatic	CNFs/chitosan	Intervertebral disc tissue engineering	CNF/chitosan formulation was injected in the intervertebral disc to restore damaged/degenerated discs	[174]
CNF	Enzymatic	CNFs/clays	Fire protection	Clay nanopaper was prepared using CNF/clay nanocomposites and evaluated for fire protection	[175,176]
CNC	Enzymatic/ mechanical	CNCs/paper	Packaging	CNCs were incorporated in paper to improve its properties	[101]
